# Spectrum of Endocrine Disorders in Central Ghana

**DOI:** 10.1155/2017/5470731

**Published:** 2017-02-23

**Authors:** Osei Sarfo-Kantanka, Fred Stephen Sarfo, Eunice Oparebea Ansah, Ishmael Kyei

**Affiliations:** ^1^Komfo Anokye Teaching Hospital, Kumasi, Ghana; ^2^Kwame Nkrumah University of Science & Technology, Kumasi, Ghana

## Abstract

*Background.* Although an increasing burden of endocrine disorders is recorded worldwide, the greatest increase is occurring in developing countries. However, the spectrum of these disorders is not well described in most developing countries. *Objective.* The objective of this study was to profile the frequency of endocrine disorders and their basic demographic characteristics in an endocrine outpatient clinic in Kumasi, central Ghana. *Methods.* A retrospective review was conducted on endocrine disorders seen over a five-year period between January 2011 and December 2015 at the outpatient endocrine clinic of Komfo Anokye Teaching Hospital. All medical records of patients seen at the endocrine clinic were reviewed by endocrinologists and all endocrinological diagnoses were classified according to ICD-10. *Results.* 3070 adults enrolled for care in the endocrine outpatient service between 2011 and 2015. This comprised 2056 females and 1014 males (female : male ratio of 2.0 : 1.0) with an overall median age of 54 (IQR, 41–64) years. The commonest primary endocrine disorders seen were diabetes, thyroid, and adrenal disorders at frequencies of 79.1%, 13.1%, and 2.2%, respectively. *Conclusions.* Type 2 diabetes and thyroid disorders represent by far the two commonest disorders seen at the endocrine clinic. The increased frequency and wide spectrum of endocrine disorders suggest the need for well-trained endocrinologists to improve the health of the population.

## 1. Introduction

Endocrine disorders account for more than 8% of the global disease burden [1]. In developing countries, however, little attention is paid to these disorders, often resulting in an increased rate of mortality and morbidity [1, 2]. This is despite the increasing numbers of noncommunicable diseases being recorded in these countries [1]. With limited resources, an increased priority is given to infectious and nutritional disorders in these countries, limiting the attention and resources dedicated towards combating endocrine and other noncommunicable disorders [3]. To worsen matters further, serious deficit exists in the number of trained endocrinologist in Sub-Saharan Africa compared to other areas of the world [4, 5]. For instance, while the estimated endocrinologist to population ratio in Sub-Saharan African countries is pegged at 0.03 per 100,000 people, those in the Americas and in Europe are 0.89 and 4.84, respectively [4, 5]. The combination of lack of skilled human resources with the absence of the needed health infrastructure for endocrinological services especially in investigative tools in developing countries means that these disorders are often missed and diagnoses delayed contributing to worsening of disability-adjusted life years in patients with these disorders [6, 7]. With this in mind, data on the practice of endocrinology from African settings is required to shed more light on the spectrum of endocrine disorders and the available level of care available for patients with these disorders. Additionally, the burden and spectrum of endocrine disorders within the community and the hospital settings need to be characterized in developing countries if strategies for improving healthcare in developing countries are to be successful. This usually involves rational setting of priorities in view of the limited resources allocated for it. In this regard, knowledge of the clinical profile of patients will assist policymakers, healthcare planners, and administrators to reach informed decisions on allocation of these resources to the various areas within the health subsector.

Although hospital-based data are inevitably referral- and access-biased, they provide substantial insight into the types of diseases, the usual age of presentation, and their burden on inpatient service [8]. In addition, they, to a great extent, reflect the morbidity pattern in the communities.

In Ghana, a West African country with a population of 25 million, there are 5 certified endocrinologists located in 3 referral hospitals in three major cities—Kumasi, Accra, and Cape Coast. In 2011, an outpatient clinic for endocrine services was opened at the Komfo Anokye Teaching Hospital, a tertiary referral center situated in Kumasi in the central belt of Ghana. The aim of this study was to profile the frequency of endocrine disorders and describe the basic demographics of patients in our endocrine outpatient service over a 5-year period.

## 2. Methods

This study was approved by the Committee on Human Research Publication and Ethics (CHRPE) of the School of Medical Sciences, Kwame Nkrumah University of Science and Technology, and the Komfo Anokye Teaching Hospital (KATH), Kumasi. This is a retrospective study conducted at the endocrine clinic of the Komfo Anokye Teaching Hospital in Kumasi, Ghana. The endocrine clinic was established in 2011 by the lead author. The clinic runs once a week and receives referrals for adults > 18 years with endocrine disorders from 6 out of the 10 administrative regions of Ghana and serves an estimated population of 10 million. A quarter of referrals to the clinic are received from health centers located within the northern and middle belts of Ghana while the remainder are referred from within the teaching hospital particularly after patients have been discharged as inpatients. The clinic has two endocrinologists (OSK and EOA) and four nurses. A review of endocrine disorders at the clinic from 2011 to 2015 was performed by a review of medical charts of patients by the two endocrinologists. Information obtained from the case files included age at presentation, gender, principal complaints, and final diagnoses. Endocrine disorders were classified according to the 10th revision of the International Statistical Classification of Diseases and Related Health Problems (ICD-10) as follows: (1) thyroid gland and thyroid hormone abnormalities, (2) disorders of the pancreas, insulin, and glucagon, (3) parathyroid gland disorders, (4) disorders of the pituitary gland, (5) disorders of the adrenal gland, (6) disorders of the gonads, and (7) other disorders of the endocrine gland. Clinical diagnoses were made by history taking and physical examination followed by laboratory and radiological studies as indicated. The range of laboratory testing available included fasting blood glucose; glycated hemoglobin; oral glucose tolerance tests; lipid profile; microalbuminuria; serum calcium; intact PTH; complete blood count; erythrocyte sedimentation rate; renal and liver function tests; thyroid profile; thyroid autoantibody test; and radiological testing including X-rays, ultrasonography, computerized tomography, and magnetic resonance imaging, where appropriate. Among the data recorded were age and gender, and these were entered into Excel spreadsheets by data entry clerks.

### 2.1. Statistical Analysis

Means and medians were compared using either the Student's *t*-test or Mann–Whitney's *U* test for paired comparisons or ANOVA or the Kruskal-Wallis tests for more than 2 group comparisons. Statistical significance was set at a two-tailed *p* value < 0.05 with no adjustment for multiple comparisons. Statistical analysis was performed using GraphPad Prism version 7 (GraphPad Software, Inc).

## 3. Results

### 3.1. Demography and Frequency of Classes of Endocrine Disorders

In 2011, 2012, 2013, 2014, and 2015, a total of 1010, 614, 574, 334, and 498 new patients, respectively, were enrolled for care at the endocrine clinic. Thus, a total of 3070 patients have been referred to the endocrine clinic between January 2011 and December 2015. This comprises 1045 males and 2086 females (male : female ratio of 1.0 : 2.0) with an overall median age of 54 (IQR, 39–69) years. The median (IQR) age of females was 53 (39–63) years while that of males was 56 (45–65) years, *p* < 0.0001. As shown in Table 1, the commonest primary endocrine disorders seen at the clinic were diabetes, thyroid disorders, adrenal disorders, and pituitary disorders at frequencies of 79.1%, 13.1%, 2.2%, and 1.4%, respectively. As shown in Table 2, the number of visits to endocrine clinic has increased from 2583 visits in 2011 to 4315 in 2015. Similarly, the proportion of visits to the OPD outfit for endocrine services increased from 4.1% to 7.2% over the period as shown in Table 2. Patients after enrolling at the clinic are followed up between one and six monthly appointments.

### 3.2. Diabetes

**Figure 1:**
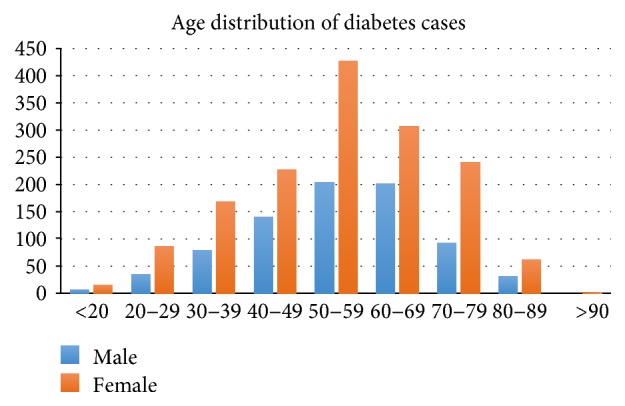
Age and gender distribution of diabetes patients at an endocrine clinic in a tertiary referral center in Ghana.

Out of 2465 patients with diabetes, 1032 were males and 1433 were females with a male : female ratio of 1.0 : 1.4. The median (IQR) age at presentation for females of 56 (45–66) years was not significantly different from that of males, 57 (47–66) years, *p* = 0.12, with an overall median age of 56 (IQR, 46–66) years. The modal age range for both males and females was 50–59 years, respectively, as shown in Figure 1. For the diabetes patients, 2261 (91.7%) had type 2 diabetes, 152 (6.2%) had type 1 diabetes, 19 (0.8%) had gestational diabetes, and 23 (0.9%) had specific type and unspecified diabetes. The median (IQR) ages of patients with type 2 diabetes, type 1 diabetes, gestational diabetes, and unspecified diabetes were 58 (49–67) years, 26 (22–32) years, 31 (24–36), and 38 (30–75) years, respectively, with *p* < 0.0001 (Kruskal-Wallis test), and the female/male proportions for T2DM, T1DM, and unspecified diabetes were 1.7 : 1, 2.3 : 1, and 1.9 : 1. Seventy-two percent of T2DM patients admitted to the clinic also had hypertension. A range of oral hypoglycemic agents including metformin, first- and second-generation sulphonylureas, and thiazolidinediones as well as different formulations of insulin are available on National Insurance Scheme for patients to access free of charge. Relatively newer agents like DPP4 inhibitors, GLP-1 agonist, amylin mimetics, and insulin analogues are not available under the health insurance scheme and are relatively expensive for patients to buy out of pocket. Antihypertensives like angiotensin-converting enzyme inhibitors, angiotensin-receptor blockers, calcium channel blockers, and centrally acting drugs like methyldopa are also on the National Health Insurance Scheme. Commonly prescribed statins include atorvastatin, rosuvastatin, and simvastatin while antiplatelet drugs such as aspirin and clopidogrel are available for cardiovascular risk reduction especially in T2DM diabetes patients. Diet therapy services are available free of charge for all patients upon admission to the clinic. All new diabetes patients are referred to the ophthalmology department for routine retinal screening and treatment of various grades of retinopathy. Yearly screening for foot and renal disorders is also in place for all patients.

### 3.3. Thyroid Gland and Hormonal Disorders

**Figure 2:**
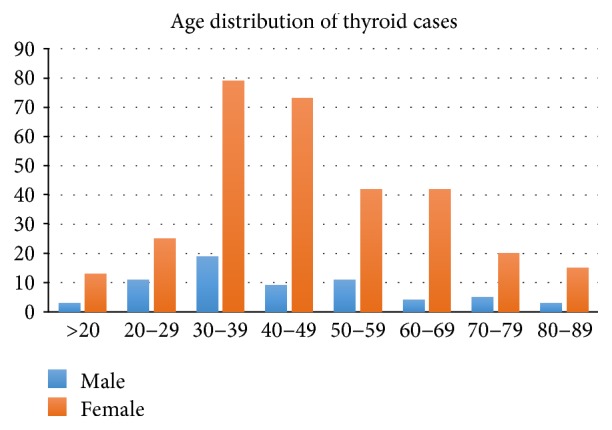
Age and gender distribution of thyroid disease patients at an endocrine clinic in a tertiary referral center in Ghana.

Four hundred and ten patients had thyroid gland and hormone disorders: 332 were females and 68 males with a male : female ratio of 1.0 : 5.0. The median (IQR) age of females was 37 (30–54), and 39 (29–52) years was for males, *p* = 0.92. The modal age of thyroid disorders was between 30 and 39 years for both sexes as shown in Figure 2. Two hundred and twenty-three (54.4%) patients were diagnosed with Graves' disease: 188 females and 35 males, with a female : male ratio of 5.4 : 1; 90 (22%) were diagnosed with toxic nodular goiter: 66 females and 24 males (female : male ratio 2.8 : 1); 18 (4.4%) had thyroiditis, 16 (3.9%) with Hashimoto's thyroiditis and 2 (0.5%) with postpartum thyroiditis; 51 (13.2%) had iodine deficiency disorders; 52 (12.7%) were diagnosed with endemic goiters; and 12 (2.9%) had thyroid malignancies. The median (IQR) ages for Graves' disease, toxic nodular goiter, thyroiditis, iodine deficiency, endemic goiters, and thyroid malignancies were 36 (28–53), 43 (40–53), 33 (28–34), 40 (25–51), 46 (27–65), and 45 (30–59), respectively, *p* = 0.01. Diagnosis for Graves' disease, toxic nodular goiter, and postpartum thyroiditis was mostly clinical with only 20% of the patients able to afford thyroid autoantibody (thyroglobulin, thyroid peroxidase) testing. In patients with Graves' disease, the first line of therapy involves the use of oral thionamides like carbimazole and methimazole. In cases of contraindications for thionamides, propylthiouracil was prescribed. Beta receptor blockade was prescribed to majority of patients with toxic symptoms. Second-line therapy involved thyroid surgery. All the patients who had total thyroidectomy developed hypothyroidism and had to be prescribed thyroxine subsequently. Antithyroid medications and their cost per month of treatment for management of Graves' disease and toxic goiters include carbimazole (≈$30–45 per month) and propylthiouracil (≈$30–60 per month). In Graves' disease patients with moderate to severe ophthalmopathy, referrals were given for ophthalmological care. Nuclear medicine services are not available at KATH. Patients with toxic thyroid disease and thyroid malignancies who require nuclear imaging and treatment are referred to Korle-bu Teaching Hospital for further management.

### 3.4. Parathyroid and Calcium Metabolic Disorders

17 patients were diagnosed with parathyroid and calcium disorders; 12 of them were females and 5 were males with a female : male ratio of 2.4 : 1. The median (IQR) age for patients with hypoparathyroid disorders was 44 (33–56) and that for patients with hyperparathyroid disorders was 45 (38–53) with *p* < 0.001. All patients with hypoparathyroidism resulted from surgical extirpation of the thyroid gland with resulting hypocalcemia. All were females with Graves' disease. Three of the patients with hyperparathyroidism had primary hyperparathyroidism from familial hypocalciuric hypercalcemia, and 2 patients had parathyroid carcinoma. The median age for those with familial hypocalciuric hyperparathyroidism was 34 (22–38) and those with parathyroid carcinoma was 28 with *p* < 0.0001. These disorders were diagnosed based on history, clinical examination, and laboratory investigations like albumin-adjusted calcium, intact PTH levels, and urinary calcium excretion rates. Parathyroid carcinoma was diagnosed based on ultrasound features with a definitive diagnosis made from histological examination of surgically removed organs. Treatment included surgery for patients who had hyperparathyroidism and, in some cases, medical therapy with oral calcium and bisphosphonates. For parathyroid carcinoma, the mainstay of therapy was surgical extirpation. Both patients, however, suffered recurrence and were transferred to Korle-bu Teaching Hospital in Accra, the capital of Ghana, for further management.

### 3.5. Pituitary Disorders

52 (2.1%) patients were diagnosed with disorders of the pituitary gland with an overall median (IQR) of 36 (27–27) and male : female ratio of 1 : 3. Of these, 61.5% had pituitary adenomas; 12 (23.1%) patients presented with acromegaly, 6 (11.5%) with Cushing's disease, and 14 (26.9%) were diagnosed with prolactinoma. Twenty-one (40.3%) presented with central diabetes insipidus. The median ages of the above disorders were 43 (35–66), 36 (32–42), 64 (52–77), and 53 (39–55), respectively, with *p* < 0.0001. Pituitary insufficiency was seen in 2 patients both 35 years old from Sheehan's syndrome. The range of medications prescribed for pituitary disorders included bromocriptine, ketoconazole, and octreotide. For patients with macroadenomas and those who failed in oral therapy, transsphenoidal hypophysectomy and radiotherapy were arranged locally and if possible internationally.

### 3.6. Adrenal Disorders

63 cases of adrenal disorders were diagnosed over the period. 20 (31.7%) had Cushing's syndrome, 4 (6.3%) had primary aldosteronism, 5 (7.9%) had adrenal carcinoma, and 4 (6.3%) had pheochromocytomas. Of those with adrenal carcinoma, 3 were females and 2 were males. The median (IQR) for those with adrenal carcinoma was 37 (36–41); for those with Cushing's syndrome, it was 35 (24–46); for Conn's syndrome, it was 42 (40–48); and for pheochromocytomas, it was 53 (53–73). There were 29 (46%) cases of adrenal insufficiency. Of patients with adrenal insufficiency, there were 13 males and 14 females with a male to female ratio of 1 : 1.1. The median age of males was 40 (32–58) and that of females was 32 (25–63), *p* < 0.0001. Fourteen of the patients with adrenal insufficiency had HIV-associated adrenalitis, and 2 had disseminated tuberculosis. The diagnosis for adrenal insufficiency involved short synacthen test; for those with Cushing's syndrome, dexamethasone suppression test was used for diagnosis with plain abdominal X-rays and CT scans used for confirmation of patients with abdominal tuberculosis. Pheochromocytomas were diagnosed mainly by histology and hormonal assay testing.

### 3.7. Gonadal Disorders

10 patients were admitted on the account of gonadal disorder. Seven males had Klinefelter's syndrome and 3 had Turner's syndrome. The mean ages were 27 (22–29) and 21 (17–26) with *p* < 0.001. Confirmation of these diagnoses was based on genetic studies, semen analysis, and testicular biopsy and supported by ultrasound studies in the selected cases.

## 4. Others

There were 63 miscellaneous diagnoses referred to the endocrine unit: six (9.5%) were for familial dyslipidemias, 45 (71.4%) for metabolic syndrome, 5 (7.9%) for multiple endocrine neoplasia type 1, 3 (4.8%) for multiple endocrine neoplasia type 2, and 4 (6.3%) for autoimmune polyendocrinopathy syndromes.

## 5. Discussion

In this cohort of 3070 patients referred to an adult endocrine specialist clinic in an urban Ghanaian setting, we found that diabetes was the commonest presenting endocrine disorder, followed by thyroid gland disorders and adrenal disorders. Globally, diabetes and thyroid disorders represent the two commonest endocrinological disorders [9–11] seen in adult endocrine specialist practice. Thus, the spectrum of endocrinological disorders in central Ghana concurs with most other studies. Most studies like this one were retrospective in design and focused on inpatient admissions. The major contributor of endocrine cases referred to the clinic was diabetes, representing 79% of the referrals. This finding is consistent with reports of an increasing prevalence of diabetes in Low- and Middle-Income Countries (LMIC) including Sub-Saharan Africa [12–16]. The rising rates of diabetes in Ghana and other Sub-Saharan African countries are reflective of the increasing longevity of their citizenry and the improvements in the socioeconomic status, leading to urbanization and adoption of Western lifestyles that are serving as undercurrents for an epidemiological transition [17, 18]. Accordingly, the population prevalence of vascular risk factors, in particular, obesity, physical inactivity, and poor dietary practices, has escalated in several countries across most of the African continent culminating in the escalating rates of cardiovascular diseases and nutritional disorders, mainly diabetes and ischemic heart diseases, on the continent [19–25]. Hence, it is not surprising that diabetes continues to be the commonest endocrine disorder in our cohort. Type 1 diabetes rates decreased over the years at our clinic in view of the establishment of a pediatric endocrinology clinic in our hospital from 2012 where adolescents with type 1 diabetes are cared for. By their care, the pediatric endocrinology clinic has access to free insulin through the Life for a Child programme instituted by the International Diabetes Federation with the aim of providing insulin to needy children and adolescents [26]. It must be noted, however, that the array of cases captured in this series was strongly influenced by the high proportions of inpatient referrals received from our teaching hospital. This could, for instance, explain the high frequency of diabetes in this cohort. Type 2 diabetes patients seen at the clinic were relatively younger compared to those in developed countries. This will increase the number of their productive years lived with the disease and its complications as well as increase the burden on the limited resources available for diabetes care. This makes it imperative for a comprehensive approach to tackling the menace of diabetes in developing countries to be instituted urgently since diabetes affects the population at the prime of their life [27, 28].

Thyroid disorders have increased in frequency with changing epidemiology from iodine deficiency-driven disorders to thyroid autoimmunity disorders in most Sub-Saharan African countries [29, 30]. This conforms to findings in our study where Graves' disease was the most prevalent thyroid disorder seen over the period of the study, contrary to previous studies conducted in Sub-Saharan Africa where iodine deficiency disorders were the most commonly observed thyroid disorders [31–33]. The changing epidemiology of thyroid diseases observed in most areas of iodization can be attributed to improvement in iodine nutrition [34]. However, unregulated iodination has beset most countries especially in low-income areas [34–36]. Ghana, since the introduction of Universal Salt Iodization in 1995, has moved from being classified as a country of severe iodine deficiency to being classified as a country of moderate iodine deficiency [37]. However, the lack of proper infrastructure to regulate iodination coupled with inadequate monitoring recently reported in some areas of the country [38] has led to a situation whereby in certain areas iodine deficiency disorders still exist, while in other areas, a transition from iodine deficiency disorders to hyperthyroid and autoimmune disorders has been observed [39]. This may explain the high number of autoimmune thyroid disorders seen in this cohort of patients. It must be added, however, that patients with euthyroid goiter and thyroid malignancies present to the surgical and eye, nose, and throat units of hospitals. It may be the case that this study undervalued the reporting of nontoxic multinodular goiters to our hospital. The Ghanaian population mostly still lags behind other regions of the world in iodine nutrition [34]. The relatively low rates of autoimmune hypothyroid disorders in our cohort in comparison with Europe and South and North America may be partly attributed to our history of iodine deficiency. Iodine sufficiency usually dictates the clinical characteristics of thyroid disorders. In areas with iodine sufficiency, as mentioned earlier, the prominent thyroid disorder is autoimmune in nature as compared to areas with iodine deficiency [39].

There was significant female gender predilection among patients with thyroid disease as previously reported with a ratio of 4 : 1 compared to males. This is similar to findings from other studies where females dominated thyroid disease incidence [34, 35]. The diagnosis and care of patients with thyroid disorders is extremely hampered by the relatively high cost of thyroid hormone profiling and imaging in our hospital. Magnetic resonance imaging and CT scans are not always available. Additionally, there is no nuclear medicine service available for use, and hence, radio imaging and radioiodine therapy are not in place for the patient's benefits. Again, the cost of antithyroid medications ranged between 30 and 50 dollars per month for patients with hyperthyroid disorders; this, when compared to an average household income of 300 dollars [40], is not encouraging since most patients will not be able to afford sustainably these medications, especially with the absence of nuclear medicine facilities, and some of these patients will have to take medications for over 18 months to be able to achieve control.

Adrenal disorders represented the 3rd commonest endocrine disorder seen in our clinic. The most common disorders as seen in other studies were Addison's disease and Cushing's syndrome [41–43]. HIV and adrenal tuberculosis have increased in prevalence with the increasing prevalence of both diseases on the continent.

Management of most of the endocrine disorders has been challenged by lack of diagnostic support for patients. CT and MRI scans are paid for by patients out of pocket, and MRI services only became available late in 2013. These limitations have impacted on our ability to establish and subclassify endocrine disorders in this cohort. Again, the absence of nuclear imaging facilities hampers both identification and treatment of adrenal as well as thyroid and parathyroid disorders. This is especially recorded in our study where there were a low number of cases of parathyroid disorders. The absence of the investigative tools requires in most instances the use of a low index of suspicion and clinical acumen to diagnose.

However, these patients were evaluated by trained endocrinologists and were mostly diagnosed on clinical grounds with laboratory confirmation in a significant proportion of cases. A further challenge in patient management is the inability to afford medications on a sustainable basis for their clinical conditions. With a median age of 54 years, patients with endocrine disorders in our cohort were either retired or unemployed due to disability; hence, enlisting endocrine care within National Health Insurance would be a major step towards alleviating the health economic burden.

A major limitation of this paper is the lack of data on disease outcomes (mortality, disease complications, etc.) which would have provided useful insights on disease burden. The study, however, has been able to show increasing numbers of patients visiting the endocrine clinic. The need for the training of more endocrinologists has become increasingly urgent to improve quality and standards of care.

## 6. Conclusion

In conclusion, within this adult urban endocrinology clinic in Kumasi, we found that noncommunicable diseases such as diabetes and thyroid and adrenal disorders contributed significantly to the burden of endocrine disorders.

## Figures and Tables

**Table 1:** Endocrine disorders in Kumasi, Ghana, according to ICD-10.

ICD-10 classification		Frequency (*n*)	Percent (%)
Pancreas, insulin, and glucagon disorders	Type 1 diabetes	152	5
	Type 2 diabetes	2261	75
	Gestational diabetes	19	0.6
	Drug induced/other forms	23	0.8

Thyroid gland and hormonal disorders	Hyperthyroidism	278	9.2
	Hypothyroidism	52	1.7
	Iodine deficiency-related disorders	51	1.7
	Thyroiditis	18	0.6
	Others	12	0.4

Parathyroid and calcium metabolic disorders	Familial hypocalciuric hyperparathyroidism	3	0.09
	Parathyroid carcinoma	2	0.07
	Primary hypoparathyroidism	7	0.2

Pituitary and hypothalamic gland disorders	Acromegaly	12	0.4
	Cushing's disease	6	0.2
	Prolactinoma	16	0.5

Adrenal disorders	Cushing's syndrome	20	0.7
	Primary hyperaldosteronism	4	0.1
	Adrenal carcinoma	5	0.2
	Adrenal pheochromocytoma	4	0.1

Gonadal disorders	Turner's syndrome	5	0.2
	Klinefelter's syndrome	3	0.1

Others	Familial hyperlipidaemia	6	0.2
	Multiple endocrine neoplasia type 1	5	0.2
	Multiple endocrine neoplasia type 2	3	0.1
	Polyendocrine autoimmune disorders	4	0.1
	Metabolic syndrome	45	1.4

Total	*N* (%)	3070	100

**Table 2:** Endocrine clinic attendance in Kumasi, Ghana, between 2011 and 2013.

Year	Total OPD attendance	Number of endocrine visits	% of OPD visits to the endocrine clinic
2011	63,335	2583	4.1
2012	65,620	4112	6.2
2013	52,400	3370	6.4
2014	59,284	3834	6.5
2015	60,322	4315	7.2
Total	300,961	18,214	6.1
